# A patient presenting with symptomatic hypomagnesemia caused by metformin-induced diarrhoea: a case report

**DOI:** 10.1186/1757-1626-2-156

**Published:** 2009-10-16

**Authors:** Anders Svare

**Affiliations:** 1 Department of Public Health and General Practice, Faculty of Medicine, Norwegian University of Science and Technology, N-7489 Trondheim, Norway; 2 Department of Medicine, Namsos Hospital, N-7800 Namsos, Norway

## Abstract

**Introduction:**

Metformin is licensed for treatment of diabetes mellitus type 2. This report describes a patient on metformin who developed diarrhoea and symptomatic hypomagnesemia. To the author's knowledge, this is the first report on metformin-induced symptomatic hypomagnesemia.

**Case presentation:**

The patient was a 57-year old Caucasian male with diabetes mellitus type 2. He had been on metformin for nine years and presented with chronic diarrhoea, spasms, paresthesias, pain, and malaise. Blood tests revealed hypomagnesemia, hypocalcemia, and hypokalemia.

**Conclusion:**

Drugs associated with diarrhoea may induce malabsorption. If malabsorption is substantial it may result in further symptoms of clinical importance. In some cases potentially life-threatening conditions may occur.

## Introduction

Metformin is a drug licensed for treatment of diabetes mellitus type 2 [1]. The use of metformin is associated with dose dependent side effects related especially to the gastrointestinal tract [1]. Diarrhoea is very common. Diabetes mellitus and diarrhoea are both common causes of hypomagnesemia [2]. Although some animal studies have shown a potential beneficial effect of metformin on magnesium status [3-5], there have been small clinical studies which have found biguanides (the drug group to which metformin belongs) to be associated with magnesium depletion [6,7].

## Case presentation

The patient was a 57-year-old Caucasian male. He was an ex-smoker and reported normal alcohol consumption. His medical history included coronary artery disease, hypertension and gastrointestinal reflux. He had been diagnosed with type 2 diabetes mellitus back in 1996. At the time he got the diabetes diagnosis, he reported that his stools for many years had tended to be a little loose. However, he described it as a minor problem, the main annoyance being the passing of loose stools once every morning. Initially he was treated with glipizide (a SU drug). Metformin was added in 1999. The dose had been gradually increased, and since 2004 he had been taking 1 g with breakfast and 0.5-1 g with supper. He had no known complications to his diabetes.

He was admitted to the Medical Ambulatory in March 2008 because of carpal and calf spasms, pain (especially in the legs), paresthesias of the lips, hands and feet, and malaise. His GP had diagnosed hypomagnesemia, hypocalcemia and hypokalemia. When seen in the Medical Ambulatory, he reported a substantial diarrhoea problem which had lasted a long time. He did not recall exactly when the diarrhoea had become a problem, but at least he remembered that it was after the introduction of metformin in 1999. When asked about any relation between the diarrhoea and the introduction of metformin or increase of metformin dose, he had not noticed any such relation. The last months before his admission in March 2008 his diarrhoea had worsened even more. These months he had been passing loose stools on average four times a day. Sometimes he had observed some mucilage in the stools. His additional medication included glimepirid (a SU drug), metoprolol, isosorbide mononitrate, ramipril, atorvastatin, clopidogrel, aspirin, and lansoprazol.

On examination his blood pressure was 192/100 mmHg. Sodium was 143 mmol/l (reference range 137-145), potassium 3.4 mmol/l (3.6-4.6), calcium 1.97 mmol/l (2.15-2.51), magnesium 0.33 mmol/l (0.71-0.94), albumin 45 g/l (36-45), creatinine 80 μmol/l (62-133), glucose 9.9 mmol/l (4.0-6.0), HbA1c 7.1% (4.0-6.0), Vitamin B12 272 pmol/l (113-603), free thyroxin 17.2 nmol/l (9.0- 19.0), TSH 2.32 mU/l (0.20- 4.5), PTH 2.3 pmol/l (1.3-7.6), 25-OH-Vitamin D 67 nmol/l (20-100), 1.25-(OH)2-Vitamin D 113 pmol/l (48-160), and anti-tissue transglutaminase antibodies IgA was negative. Thus, the blood tests showed hypomagnesemia, hypocalcaemia and hypokalemia.

It was concluded that he suffered from severe hypomagnesemia, most likely due to diarrhoea caused by metformin. Therefore, metformin was discontinued and daily intake of magnesium and potassium was instituted. He reported that the consistency of his stools improved markedly in the course of a few days after the discontinuation of metformin.

At a follow-up consultation three weeks later, magnesium was 0.80 mmol/l, calcium 2.49 mmol/l, albumin 43 g/l and potassium 4.4 mmol/l. However, his blood glucose was found to be unacceptably high. The glimepiride dose was increased, and he also started to take sitagliptin. In addition, he was suggested to try a reduced metformin dose, 0.5-1.0 g daily. When seen again in August 2008, he was still not feeling well. Because of diarrhoea he had stopped taking metformin a few days after the reinstitution of the drug, and again the stools' consistency had improved rapidly. His plasma glucose was 10-20 mmol/l. Therefore, the oral antidiabetic drugs were discontinued and a two-dose NPH insulin regime was instituted. At a follow-up consultation in December 2008 his diabetes was well regulated, serum electrolyte levels were within the normal range and loose stools now was a minor problem. About two months before this consultation the patient, without consulting any physician, had discontinued the magnesium and potassium tablets.

When seen last time in September 2009, he still was in good shape and with only minor bowel problems. His blood tests were all within the normal range: magnesium 0.99 mmol/l, sodium 142 mmol/l, potassium 4.1 mmol/l, calcium 2.40 mmol/l, creatinine 102 mmol/l, and albumin 45 g/l.

## Discussion

The electrolyte deficiencies described in this case are assessed to be the result of metformin-induced chronic diarrhoea. The Naranjo algorithm is a questionnaire used to assess whether symptoms or findings are actually adverse effects of specific drugs, or caused by other factors[8]. Scores range from -3 to +12. A score of 5-8 classifies the symptoms or findings as probable adverse drug effects, whereas a score ≥ 9 describes a definite adverse drug effect. When the Naranjo algorithm is applied to this patient, using the combination of hypomagnesemia and severe diarrhoea as the adverse effect, the following scores are obtained: These adverse effects have been reported before (+1). The adverse effects appeared after metformin was given (+2). The diarrhoea and hypomagnesemia improved after metformin was discontinued (+1). The diarrhoea (and presumably the hypomagnesemia) reappeared when metformin was tried again later (+2). The electrolyte deficits were objectively verified (+1). At last, there is the question whether the symptoms and findings were definitely caused by the drug, or if there are alternative explanations. Diabetes may cause hypomagnesemia[2]. Besides, even before he got the diabetes diagnosis, he reported his stools to be loose, and diarrhoea unrelated to metformin could also be causal [2]. At last, there are many other theoretical causes, including his other drugs [2]. However, based on the HbA1c, his diabetes was well regulated at the time of his admission. His diabetes treatment was changed during the months after the metformin discontinuation. Glimepiride was increased, sitagliptin was tried for a few months, and from August the patient was put on NPH insulin in monotherapy. A small study from 1988 showed a beneficial effect of glipizide on serum magnesium in poorly regulated type 2 diabetics [7]. However, the patient had been on SU drugs for a long time at his admission, and it is very unlikely that the dose increment caused the serum magnesium improvement. In a study from 1992, neither insulin nor SU drugs improved hypomagnesemia in diabetes mellitus type 2 patients [9]. This should also rule out insulin treatment as the probable cause of improvement. No reports on the effect of sitagliptin or related drugs on magnesium status exist, and the short spell it was used, makes an effect highly unlikely. His stools were still a little loose after the discontinuation of metformin, but the electrolytes remained inside the reference range. Diarrhoea not caused by metformin is then also an unprobable cause. As no other interventions than a change in diabetes treatment and temporary treatment with magnesium and potassium were done, potential side-effects of his other drugs can be ruled out. At last, even if all other theoretical causes have not been investigated, the substantial improvement after the metformin discontinuation makes other explanations highly unlikely. The refusal of alternative explanations gives a Naranjo algorithm score of +2, and a total score of +9. This then, according to the Naranjo algorithm, definitely defines metformin as the cause of the diarrhoea and electrolyte deficits.

The patient's symptoms were caused by the electrolyte deficits, of which hypomagnesemia was the most important. Hypomagnesemia is a common cause of hypocalcemia.

After discontinuation of metformin his blood glucose and HbA1c increased to unacceptable levels. This occurred despite the use of other oral antidiabetic agents. Therefore, insulin had to be instituted. The author presumes that metformin, apart from the antidiabetic effect per se, also may have caused some malabsorption of carbohydrate, since HbA1c was 7.1% when the patient was initially seen.

Metformin has been known since the 1950s, and its use has increased over the last 20 years. Today it is a drug of choice for the treatment of diabetes mellitus type 2. In the USA, it is one of the most prescribed drugs, with over 34 million prescriptions for generic metformin alone in 2006 [10]. Metformin is commonly associated with gastrointestinal side effects, such as diarrhoea, flatulence and abdominal discomfort [1]. Approximately 5% of patients who start taking metformin do not tolerate these side effects. Due to malabsorption about one in five patients develops vitamin B12 deficiency. However, the vitamin deficiency is usually not of clinical significance. Apart from this deficiency, reports on nutritional deficits caused by metformin are rare. One reason may be that studies just report deficits associated with diarrhoea in general, without emphasizing the cause of the diarrhoea.

Diabetes mellitus is a risk factor for magnesium depletion [2]. Studies in animals suggest a beneficial effect of metformin per se on magnesium status. In diabetic rats, metformin was shown to improve the concentration of liver magnesium, but no effect on serum magnesium was described [3,4]. In diabetic patients on metformin, intracellular magnesium levels measured in peripheral monocytes were found to be substantially higher than in patients not receiving metformin [5]. However, in a study using cultured human peripheral blood monocytes, exposure to metformin was not associated with high intracellular content of magnesium [11]. In a study on 34 poorly regulated diabetes mellitus type 2 patients with hypomagnesemia from 1988, treatment with glipizid improved the serum magnesium status, whereas metformin treatment had no effect [7]. However, this study only reported on-treatment data, while reporting intention-to-treat data is generally considered the correct method today. A study from 1979 measured serum magnesium in a mixture of 582 type 1 and type 2 diabetics [6]. Multiple regression analyses showed treatment with biguanides to be associated with lower serum magnesium than sulphonylurea-based therapy. However, only 36 patients were treated with biguanides, and the biguanide most frequently used was not metformin but phenformin. To sum up, the relation between metformin and magnesium has previously been very infrequently studied, and no definite conclusions can be drawn. Further research should aim at elucidating this topic.

## Conclusion

This report presents a patient where the use of metformin caused chronic diarrhoea which subsequently led to severe hypomagnesemia. To the author's knowledge, this is the first report of symptomatic hypomagnesemia caused by metformin-induced diarrhoea. Several medications are associated with gastrointestinal side effects such as diarrhoea. Usually the side effects do not induce clinically relevant deficits. However, the physician should bear in mind that diarrhoea may cause severe and potentially life-threatening malabsorption.

## Abbreviations

GP: general practitioner; PCI: percutaneous coronary intervention; PTH: parathyroid hormone; NPH: neutral protamine Hagedorn; SU: sulphonylurea; TSH: thyroid stimulating hormone.

## Competing interests

The author declares that he has no competing interests.

## Authors' contributions

AS was responsible for the treatment of the patient and wrote the manuscript.

## Consent

Written informed consent was obtained from the patient for publication of this case report. A copy of the written consent is available for review by the Editor-in-Chief of this journal.
